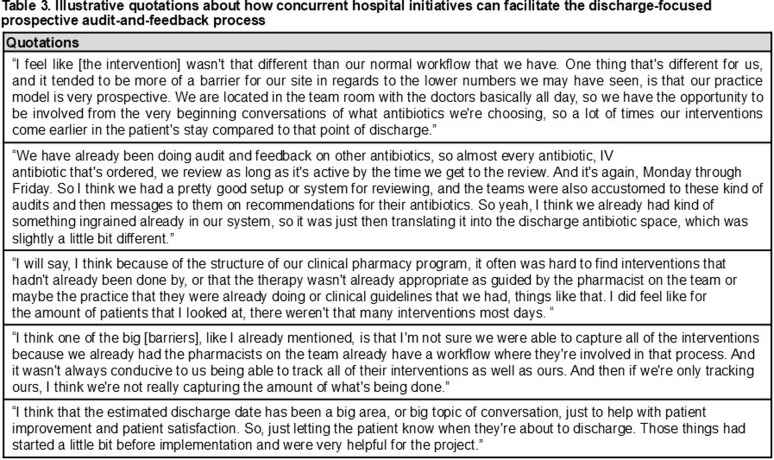# 71 Oral Vancomycin in the treatment of Clostridioides difficile Infection: a single-centre observational study in Southern Poland

**DOI:** 10.1017/ash.2026.10501

**Published:** 2026-06-23

**Authors:** DeShauna Jones, Emily Chasco, Daniel Livorsi

**Affiliations:** 1 University of Iowa; 2 Institute for Clinical and Translational Science, University of Iowa; 3 University of Iowa, Department of Internal Medicine

## Abstract

**Background:**?It is unclear how antibiotic stewardship programs can effectively address antibiotic overuse at hospital discharge. A recent stepped-wedge cluster-randomized clinical trial evaluated the effectiveness of performing prospective audit-and-feedback on patients as they approached hospital discharge. In this study, we assessed barriers and facilitators to performing this intervention during the clinical trial. Methods: We performed a qualitative study using semi-structured interviews across ten acute-care hospitals participating in the trial, including three Veteran’s Health Administration (VA) hospitals, two academic medical centers and five community hospitals (Table 1). We created a semi-structured interview guide based on the RE-AIM framework to focus on perceptions of implementing the intervention. We interviewed 14 antibiotic stewardship personnel. All interviews were audio recorded, transcribed, and coded in a three-person team. Using thematic analysis, codes were developed and collapsed into themes. **Results:** Half of the intervention sites struggled to identify patients at discharge, limiting the stewardships teams’ ability to conduct prospective audit-and-feedback at discharge (Table 2). In contrast, strong provider-stewardship relationships and existing hospital initiatives, such as handshake stewardship and discharge rounds, facilitated implementation of the intervention (Table 3). Stewardship teams at four sites also reported challenges guiding antibiotic use for patients who had formal Infectious Disease consults, as prescribers typically preferred to follow the documented recommendations from the ID team. **Conclusions:** Our findings underscore the importance of accounting for the hospital and organizational context when implementing discharge-focused audit-and-feedback interventions, paying particular attention to existing policies, procedures, and the dynamics between antibiotic stewardship teams and front-line prescribers.

**Figure:**
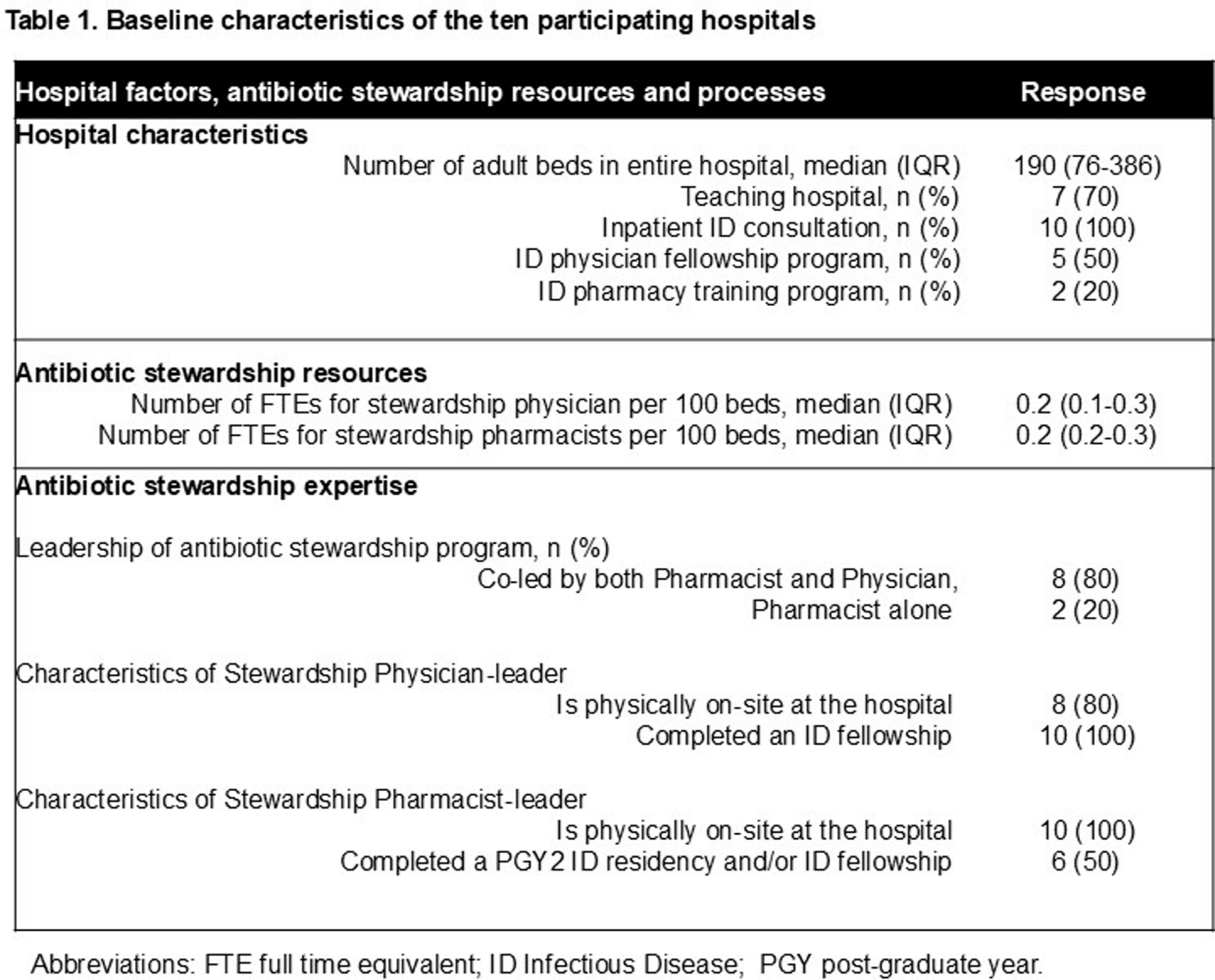

**Figure:**
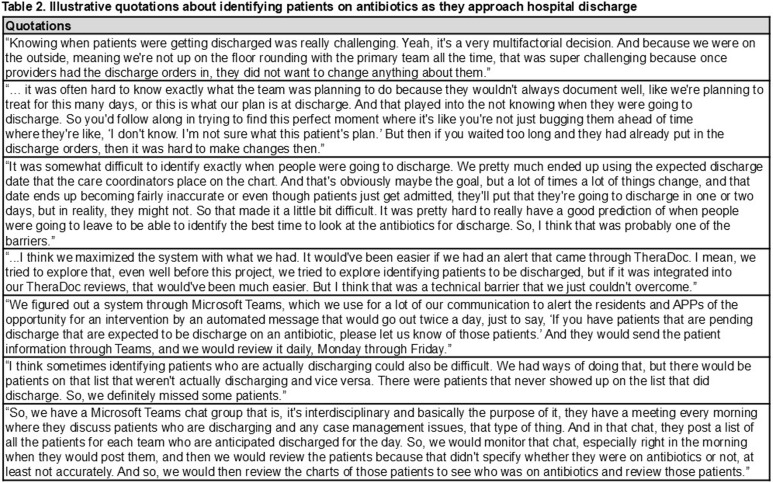

**Figure:**